# The Roles of circRNAs in Intervertebral Disc Degeneration: Inflammation, Extracellular Matrix Metabolism, and Apoptosis

**DOI:** 10.1155/2022/9550499

**Published:** 2022-02-11

**Authors:** Hao Li, Lijun Tian, Jianhua Li, Yongjin Li, Lilong Du, Zhenxin Huo, Baoshan Xu

**Affiliations:** ^1^Department of Minimally Invasive Spine Surgery, Tianjin Hospital, No. 406, Jiefangnan Road, Hexi District, Tianjin, China 300211; ^2^Graduate School, Tianjin Medical University, 22 Qixiangtai Road, Tianjin, China; ^3^Department of Orthopaedics, The First Affiliated Hospital of Baotou Medical College, Inner Mongolia University of Science and Technology, Baotou, Inner Mongolia, China 014010; ^4^Department of Orthopaedics, Tianjin Haihe Hospital, Tianjin 300350, China

## Abstract

Low back pain (LBP) is seriously harmful to human health and produces heavy economic burden. And most scholars hold that intervertebral disc degeneration (IDD) is the primary cause of LBP. With the study of IDD, aberrant expression of gene has become an important pathogenic factor of IDD. Circular RNAs (circRNAs), as a kind of noncoding RNA (ncRNA), participate in the regulation of genetic transcription and translation and further affect the expression of inflammatory cytokine, metabolism of extracellular matrix (ECM), the proliferation and apoptosis of cells, etc. Therefore, maybe it will become a new therapeutic target for IDD. At present, our understanding of the mechanism of circRNAs in IDD is limited. The purpose of this review is to summarize the mechanism and related signaling pathways of circRNAs in IDD reported in the past. Particularly, the roles of circRNAs in inflammation, ECM metabolism, and apoptosis are emphasized.

## 1. Introduction

Low back pain (LBP) is one of the most common symptoms of orthopedic patients all over the world and causes a heavy burden [1–3]. According to the Global Burden of Diseases, Injuries, and Risk Factors Study 2017 (GBD 2017), low back pain was regarded as the primary cause of years lived with disability (YLDs) counts in 2017 [4]. Besides, a systematic analysis for the GBD 2019 indicated that low back pain had the highest need of rehabilitation services in 134 of the 204 countries and brought serious economic burden [5]. From the etiology of LBP, intervertebral disc degeneration (IDD) is mainly responsible for the LBP and is a key target for the diagnosis and therapy of LBP [6–8]. It is well known that aging, trauma, and genetic predisposition may change the function and structure of the nucleus pulposus cells (NPCs), thus disc degeneration and pain [9, 10]. Previously, the chief suspected risk factor for intervertebral disc degeneration was heavy physical loading, which was commonly viewed as a wear-and-tear phenomenon [11]. However, at present, more researches suggested that IDD is genetically driven [11–14]. They found that there are many significantly differentially expressed genes (DEGs) in IDD tissues compared with normal tissues [15–18]. Among this, DEGs, noncoding RNAs (ncRNAs), as an important regulatory element, play critical roles in IDD [19]. Therefore, we suppose that these ncRNAs will provide a new direction for diagnosis and treatment of IDD. More recently, the reports of circular RNAs (circRNAs) add fuel to the ncRNA research and indicate that circRNAs can regulate pathological process of IDD as competing endogenous RNAs (ceRNAs), which include inflammation, ECM metabolism and NPC proliferation, autograph, and apoptosis [20–22]. However, at present, the mechanism of circRNAs is not completely clear. The researches of circRNAs have great potential and may become a new target for the treatment of IDD.

## 2. The Mechanism of Intervertebral Disc Degeneration

The central nucleus pulposus (NP), peripheric annulus fibrosus (AF), and cartilaginous end plates (CEP) constitute the intervertebral disc (IVD), which maintain the structure and function of the spine [23].

The IVD includes multiple collagen types, chief among them are type I and type II collagen [24], and there is a gradual transition from type II to type I from the central nucleus pulposus to the peripheral annulus fibrosus [25]. The disc possesses a variety of proteoglycans (PGs) in its extracellular matrix (ECM), and aggrecan is the most abundant proteoglycan in the disc, which is responsible for maintaining the moisture and mechanical load of IVD and is precondition for IVD to exert its physiological function and absorb stress [24, 26–28]. Proteoglycans can make the NP highly hydrated with high osmolarity, enabling the IVD to buffer pressure loads and to maintain morphology [29–31]. Early in the disc degeneration, the nucleus pulposus loses proteoglycans, leading to the decline of osmotic pressure in IVD [32]. This affects its biomechanical function [30]. The correlation study suggested that the proteoglycan content positively correlated with the viscoelastic properties of the disc; however, there was no correlation with the collagen content. These results suggest that the proteoglycan play a dominant role for maintaining the properties of IVD [29]. The AF is composed of multiple concentric annulus, resisting the stress in all directions [33, 34]. The cartilage endplate is a hyaline cartilage located on the upper and lower part of the intervertebral disc [35]. It is important that the nutrients of the NP cells mainly come from the microvascular system in the cartilage endplate, which passes through the endplate and then spreads through the nucleus pulposus matrix [36, 37]. However, there is almost no vascular supply in the adult disc. The intervertebral disc is also regarded as the largest avascular tissue in adults [38, 39].

**Figure 1:**
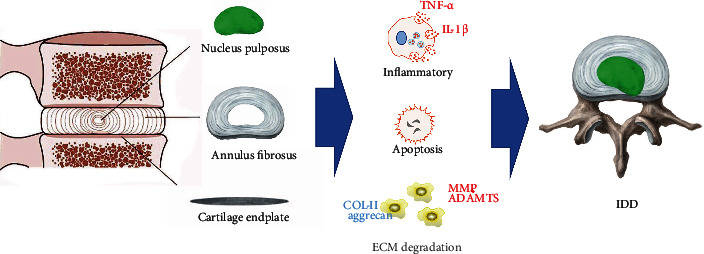
The component of intervertebral disc and the major pathologic process of degeneration.

Currently, people's understanding of IDD is limited. However, there are abundant evidences indicated that various factors, such as genetic factors, mechanical stress, trauma, fatty, and smoking, are associated with the pathomechanism of IDD [31]. Finally, the above factors disturb homeostasis, changing the morphology and function of the IVD and ultimately resulting in herniation and pain [40]. During the process, degradation of the ECM, inflammation, and apoptosis play a dominant role [41] (Figure 1). Significantly, circRNAs, as a kind of noncoding RNAs, participate in the occurrence and development of IDD [19, 21, 42–44]. The purpose of this review is to summarize the types and functions of circRNAs involved in the pathological process of intervertebral disc. We also speculated that circRNAs may become a new target for the diagnosis or treatment of IDD in the future [45, 46].

## 3. The Characteristic of circRNAs

ncRNAs are important regulatory elements and play critical roles in diverse diseases [19, 47, 48], which include microRNAs (miRNAs), long ncRNAs (lncRNAs), and the recently discovered circRNAs [49, 50]. Sanger et al. first discovered that circRNAs are a kind of ncRNAs with high thermal stability in plant-infected virions in 1976 [51]. circRNAs have no free 3′ or 5′ end, which forms a closed loop structure with 5′ and 3′ ends joining together [52, 53]. Currently, in terms of the type and quantity of the parental gene, many scholars have identified seven types of circRNAs, and exonic circRNAs are the most common type of circRNAs [51]. With increasing researches, circRNAs are considered to have several possible functions, including binding to mRNA competitively and regulating genetic transcription and translation [20, 22, 42, 52, 54]. With the deepening of the study of circRNAs, the roles of circRNAs in the occurrence and development of the disease have been gradually recognized [55–57]. It could even serve as a marker for disease diagnosis [58–60]. In the meantime, increasing evidences show that circRNAs are also closely associated with the process of IDD. Zhang et al. [19] investigated a series of public datasets (GSE67566, GSE56081, and GSE63492) and identified 586 circular RNAs that were expressed differently in IDD compared with normal discs. Gene Ontology (GO) analysis demonstrated that these differently expressed circRNAs were involved in the regulation of cellular component, gene expression, and metabolic processes. Wang et al. [61] also identified 7294 circRNAs significantly differently expressed in degenerated human NPCs by microarray analysis. Besides, with the innovation of technology, the function of circRNAs has been gradually verified in the cellular and molecular level.

## 4. The Roles of circRNAs in Intervertebral Disc Degeneration

### 4.1. Inflammation

The increase in levels of the inflammatory cytokines is the character of IDD [62]. Numerous studies also have revealed higher expression of the proinflammatory cytokines TNF-*α*, IL-1*α*, IL-1*β*, and IL-6 in degenerative disc [6, 63–69]. These cytokines promote ECM degradation, chemokine production, and change of the phenotype of cells [70–72], finally leading to the degeneration of IVD, as well as disc herniation and radicular pain [6].

**Figure 2:**
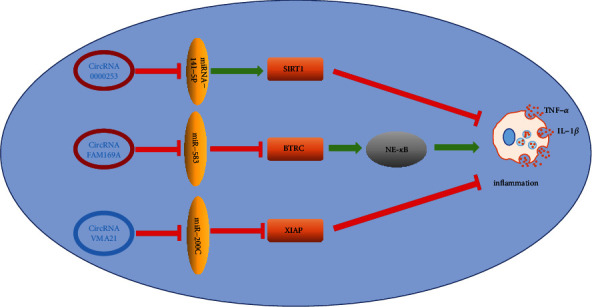
Among the reported circRNA, three kinds of circRNA play an important role in intervertebral disc inflammation, among which circRNA0000253 and circRNA FAM169A can promote the synthesis and secretion of intervertebral disc inflammatory cytokines, promoting the occurrence of inflammatory reaction and promoting intervertebral disc degeneration. On the other hand, circRNA VMA21 can inhibit the inflammatory reaction of intervertebral disc and protect the intervertebral disc to a certain extent.

Recently, increasing evidences indicated that circRNAs are correlation with the production of inflammatory cytokines. Song et al. [73] studies have shown that circRNA_0000253 can be used as ceRNA to combine with miRNA-141-5p, thus promoting the synthesis and secretion of IL-1*β* which stimulate oxidative stress response and expression of apoptotic proteins such as caspase3/7/9 and promote the expression of matrix proteases (MMP-3 and ADAMTS5), while inhibit the synthesis of COL-II and aggrecan. In addition, Guo et al. [44] found that the competitive binding of circRNA FAM169A and miRNA-583 promoted the expression of BTRC, thus promoting the secretion of inflammatory factors. On the contrary, Heng et al. [74] studies have shown that circRNA VMA21 inhibited the synthesis and secretion of inflammatory cytokines by binding miRNA-200C and promoted the expression of X-linked inhibitor of apoptosis protein (XIAP), while XIAP can bind and inhibit the activity of the apoptosis-related protein, particularly caspase3/7/9 [75, 76]. In addition to the role of caspase inhibition, an increasing number of evidence indicated that XIAP can regulate inflammation. Downregulation of XIAP facilitates the proinflammatory effect of TNF-*α* and excessive IL-1*β* secretion, causing severe sterile inflammation [77–81]. The etiology of intervertebral disc degeneration is multigenic. However, an increasing body of evidence showed that excessive secretion of inflammatory cytokines is the chief factor in IDD [9, 82] (Figure 2).

### 4.2. ECM Metabolism

**Figure 3:**
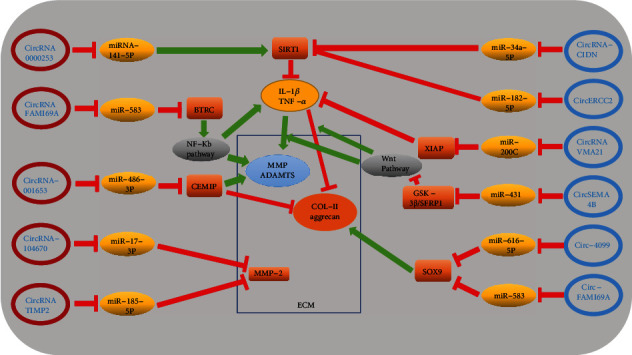
Red-labeled circRNA has a negative effect on the metabolism of extracellular matrix and can promote the catabolism of ECM. However, blue-labeled circRNA plays a role in promoting the anabolism of ECM. Song et al. [73] suggested that circRNA_0000253 could promote IDD by adsorbing miRNA-141-5p and downregulating SIRT1. Guo et al. [44] revealed that circ-FAM169A promotes IDD development via the miR-583/BTRC signaling pathway. However, Li et al. [87] proved that circ-FAM169A promote NPC proliferation and extracellular matrix synthesis by the circ-FAM169A-miR-583 pathway. Cui and Zhang [88] suggested that circ_001653 inhibited ECM synthesis of NPCs in IDD by the miR-486-3p/CEMIP axis. Song et al. [89] proved that circRNA_104670 is upregulated in human IDD tissues and upregulates MMP-2 by directly sponging miR-17-3p. Guo et al. [90] revealed that circ-TIMP2 promoted dysmetabolism of ECM via the miR-185-5p-MMP2 pathway. Xiang et al. [91] revealed that circRNA-CIDN downregulated the expression of ECM catabolism enzymes (MMP-3 and MMP-13) and upregulated the level of anabolism markers (collagen II and aggrecan) by the miR-34a-5p/SIRT1 pathway to retard compression-induced ECM degradation. Xie et al. [52] demonstrated that circERCC2 can mitigate ECM degradation via targeting miR-182-5p/SIRT1 axis. Cheng et al. [74] revealed that circVMA21 could alleviate inflammatory cytokine-induced dysmetabolism of ECM through the miR-200c-XIAP pathway. Wang et al. [92] demonstrated that circSEMA4B acts as a sponge and a ceRNA form miR431 in NPCs and competes with GSK-3*β* and SFRP1 for miR-431 binding, thus inhibiting IL-1*β*-induced degenerative process in NPCs through Wnt signaling. Wang et al. [84] revealed that circular RNA circ-4099 regulates ECM synthesis by blocking miR-616-5p inhibition of Sox9.

ECM is a noncellular, complex, and highly dynamic structure, regulating cellular function, facilitating communication between diverse cells, and maintaining homeostasis [83]. Among its complex component, aggrecan and collagen are crucial for its integrity and function [84]. However, matrix metalloproteinases (MMPs) and a disintegrin and metalloproteinases with thrombospondin motifs (ADAMTS) are closely related with ECM degradation [61, 81]. Similarly, accumulating studies have shown that some circRNAs are involved in the regulation of ECM metabolism. Up to now, 10 circRNAs are reported that are related to ECM metabolism, of which 5 circRNAs promote ECM catabolism (circRNA_0000253, circRNA TIMP2, circRNA-001653, circRNA-CIDN, and circRNA-104670). On the contrary, there are 4 circRNA (circRNA VMA21, circERCC2, circSEMA4B, and circ-4099) promoted the anabolism of ECM (Figure 3). However, the role of circRNA FAM169A may remain controversial. Inflammatory cytokines can upregulate the production of catabolic factors such as MMPs to facilitate the degradation of ECM [85, 86]. Therefore, Guo et al. [44] revealed that circ-FAM169A regulate NF-*κ*B pathway-induced IL-1*β* and TNF-*α* production via the miR-583/BTRC signaling pathway to upregulate the expression of MMP-13 and ADAMTS-5 and downregulate the expression of collagen II and aggrecan to promote IDD. However, Li et al. [87] proved that circ-FAM169A alleviate IDD development by promoting NPC proliferation and extracellular matrix synthesis via the circ-FAM169A-miR-583 pathway. In their study, they believe that miR-583 can bind to downstream mRNA such as MMP2, insulin-like growth factor 1 (IGF1), and SRY-related high mobility group box 9 (Sox9) possibly to regulate the metabolism of ECM, NPC apoptosis, and proliferation. Overall, the above two different results show that one RNA may play opposite roles in the development of IDD. This result may be due to the different stages of disease development, the inherent dual role of circRNAs, and the limitations in the understanding. Until now, almost all known circRNAs are involved in regulating the metabolism of ECM, indicating that ECM metabolism disorder may be an intermediate process in the pathological mechanism of IDD, and promoting ECM synthesis by circRNA may delay or even reverse the development of IDD, which may be a new breakthrough in the diagnosis and treatment of IDD in the future.

### 4.3. Apoptosis

**Figure 4:**
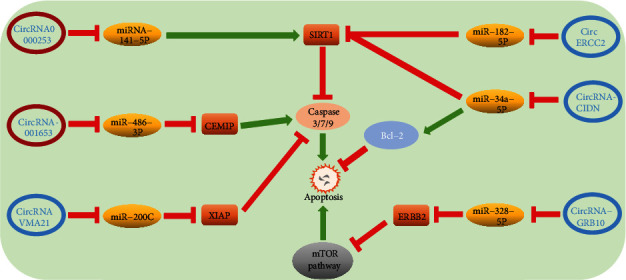
(a) CEMIP: cell migration-inducing hyaluronan-binding protein is closely related to cellular invasion, proliferation, and motility [88] (b) XIAP: XIAP belongs to the inhibitor-of-apoptosis proteins (IAP) that represent a family of endogenous caspase inhibitors and can bind and directly inhibit the activity of the three most important apoptosis effector caspases: caspase-3, caspase-7, and caspase-9 [74]. (c) ERBB2: ERBB2 is tyrosine kinase receptor which inhibits autophagy via the formation of a complex with Beclin 1, which is a key regulator of autophagy [82]. (d) SIRT1 is a NAD^+^-dependent deacetylase that can reduce apoptosis in several different cells [73]. (e) Bcl-2 belongs to the antiapoptotic protein [91].

Currently, programmed cell death (PCD) and necrosis are the main forms of cell death. Apoptosis, also known as type I PCD, is featured by chromosomal concentration, cell shrinkage, DNA degradation, and apoptotic body formation and relies on caspase [93]. It causes continuous cell loss throughout life and is closely associated with the degenerative diseases [94–96]. Recently, several studies pay attention to the relationship between circRNA and apoptosis of NP cells (Figure 4). For example, Song et al. [73] have shown that circRNA_0000253 was confirmed to facilitate IDD by inhibiting miRNA-141-5p and downregulating SIRT1, thus increasing the expression of apoptosis-related proteins such as caspase3/7/9 to promote apoptosis. Caspases are a protein family that plays a crucial role in regulating cell apoptosis (caspase-3/6/7/8/9 in mammals) and inflammation (caspase-1/4/5/12 in humans and caspase-1/11/12 in mice) [97]. SIRT1 is a highly conserved nicotinamide adenine dinucleotide- (NAD-)^+^ dependent lysine deacetylase and has been related with longevity and lifespan extension, which widely involved in signaling pathways of inflammation, cell proliferation, and death [98, 99]. Except that the circRNA-miRNA-SIRT1-caspase pathway has been proved regulating apoptosis of NPCs many times [52, 91], Cui and Zhang [88] suggested that circ_001653 could bind miR-486-3p, facilitating the expression of downstream CEMIP and caspase3/7/9, increasing NPC apoptosis. On the contrary, Cheng et al. [74] revealed that circular RNA VMA21 protects against IDD. Circular RNA VMA21 target miR-200c and XIAP to alleviate caspase-induced NPC apoptosis. Besides, there are also reports that show overexpressed circ-GRB10 inhibit miR-328-5p to upregulate the expression of ERBB2 to alleviate apoptosis of NPCs via the mTOR pathway [82]. Therefore, it seems feasible to alleviate IDD by circRNA to alleviate NPC excessive apoptosis.

## 5. Conclusion

IDD is seriously harmful to human health, but the pathomechanism of IDD is not fully understood. With people's understanding of disease, the influence of gene on disease has become a new research hotspot, and increasingly, scholars believe that genetic factor is one of the most important causes of IDD. circRNAs, as a kind of ncRNAs, can bind to the target miRNAs to regulate gene replication, transcription, and translation. With the deepening of research, there are more evidences indicated that circRNAs play an important role in the regulation of inflammation, NPC apoptosis and ECM metabolism, etc. (Table 1). Inflammation plays a critical role in the development of IDD. The overexpression of proinflammatory cytokines and the onset of an inflammatory environment induce the cascade of degenerative events that may eventually cause pain. The current treatment of IDD also mainly focuses on the control and elimination of local inflammatory response to relieve symptoms [100–102]. Therefore, it is very promising that circRNA regulates the levels of inflammatory factors and related proteins to relieve pain and delay the development of IDD. Besides, almost all known circRNAs are involved in regulating the metabolism of ECM. We speculate that ECM metabolism disorder may be an intermediate process in the pathological mechanism of IDD. Consequently, intervening the progress of IDD by regulating the metabolism of ECM at the level of genes seems to be a more promising option. However, we still have a lot of confusion about the circRNAs. For example, in the etiology of IDD, how to determine the key circRNAs? In the development of IDD, does different key circRNAs play a leading role at different stages? What factors affect circRNAs in the process of participating in the development of the disease? Therefore, the relationship between circRNAs and disease still needs to be further explored, and we speculate that circRNAs may become indicators of early diagnosis for IDD and a new target for preventing, delaying, or even reversing the pathological process of IDD at the genetic level in the future.

## Figures and Tables

**Table 1:** The role of circRNAs in intervertebral disc degeneration.

circRNA	Pathway	Function	Reference
circRNA_0000253	miRNA-141-5p/SIRT1	Apoptosis/inflammation/ECM catabolism	[73]
circRNA VMA21	miR-200c/XIAP	Anti-inflammation/antiapoptosis/ECM anabolism	[74]
circRNA-GRB10	miR-328-5p/ERBB2	Antiapoptosis	[82]
circRNA TIMP2	miR-185-5p/MMP2	ECM catabolism	[90]
circRNA-001653	miR-486-3p/CEMIP	Apoptosis/ECM catabolism	[88]
circRNA FAM169A	miR-583/BTRC	Inflammation/ECM catabolism	[44]
circRNA FAM169A	miR-583/Sox9	Antiapoptosis/ECM anabolism	[87]
circRNA-CIDN	miR-34a-5p/SIRT1	Antiapoptosis/ECM anabolism	[91]
circERCC2	miR-182-5p/SIRT1	Antiapoptosis/ECM anabolism	[52]
circSEMA4B	miR-431/GSK-3*β*/SFRP1	ECM anabolism	[92]
circRNA-104670	miRNA-17-3p/MMP2	ECM catabolism	[89]
circ-4099	miR-616-5p/Sox9	ECM anabolism	[84]
